# The OLYMPUS Architecture—Oblivious Identity Management for Private User-Friendly Services

**DOI:** 10.3390/s20030945

**Published:** 2020-02-10

**Authors:** Rafael Torres Moreno, Jorge Bernal Bernabe, Jesús García Rodríguez, Tore Kasper Frederiksen, Michael Stausholm, Noelia Martínez, Evangelos Sakkopoulos, Nuno Ponte, Antonio Skarmeta

**Affiliations:** 1 Department of Information and Communications Engineering, University of Murcia, 30100 Murcia, Spain; rtorres@um.es (R.T.M.); jesus.garcia15@um.es (J.G.R.); skarmeta@um.es (A.S.); 2 Security Lab, Alexandra Institute, N. 8200 Aarhus, Denmark; tore.frederiksen@alexandra.dk (T.K.F.); michael.stausholm@alexandra.dk (M.S.); 3 Consulting and Development, Logalty, 08036 Barcelona, Spain; noelia.martinez@logalty.com; 4 Scytales AB, 187 66 Täby, Sweden; sakkopul@unipi.gr; 5 Multicert, 4100-468 Porto, Portugal; nuno.ponte@multicert.com

**Keywords:** privacy, security, identity management, IoT, digital identities, identity derivation, oblivious cryptography

## Abstract

Privacy enhancing technologies (PETs) allow to achieve user’s transactions unlinkability across different online Service Providers. However, current PETs fail to guarantee unlinkability against the Identity Provider (IdP), which becomes a single point of failure in terms of privacy and security, and therefore, might impersonate its users. To address this issue, OLYMPUS EU project establishes an interoperable framework of technologies for a distributed privacy-preserving identity management based on cryptographic techniques that can be applied both to online and offline scenarios. Namely, distributed cryptographic techniques based on threshold cryptography are used to split up the role of the Identity Provider (IdP) into several authorities so that a single entity is not able to impersonate or track its users. The architecture leverages PET technologies, such as distributed threshold-based signatures and privacy attribute-based credentials (p-ABC), so that the signed tokens and the ABC credentials are managed in a distributed way by several IdPs. This paper describes the Olympus architecture, including its associated requirements, the main building blocks and processes, as well as the associated use cases. In addition, the paper shows how the Olympus oblivious architecture can be used to achieve privacy-preserving M2M offline transactions between IoT devices.

## 1. Introduction

Current Identity Management (IdM) systems still fail to truly fulfil security and privacy management requirements, such as unlinkability of users across service providers (SP), hiding SPs from Identity Providers (IdP), selective disclosure of personal data, usability and performance.

In this regard, traditional Single Sign On (SSO) [1] approaches for IdM, based on extended technologies such as OAuth [2] or SAML [3], have introduced significant issues regarding the management of private information in a reliable manner and are still unresolved. Web services typically verify email addresses or phone numbers using single-use codes, at best. However, age verification, a common check given the amount of restricted online resources available, is often done by merely verifying that the user owns a credit card. This system, although widespread, causes credit cards to be used for a purpose for which they were not originally intended.

In this context, governments and other official bodies started to issue electronic identities in an attempt to remedy these situations. This is usually done through the expedition of physical electronic identification cards in smart-card format, for later use in devices such as smartphones, laptops, and so forth. However, this approach raises other problems. For example, electronic identities of different governments or organizations are often incompatible with each other so that service providers are forced to choose what types to support and to maintain complex infrastructures for that purpose. European projects such as STORK [4] represent a step forward to the use of digital identity between countries while serving as a precedent for eIDAS regulation [5].

Thus, despite efforts to deploy digital identity systems, user and password systems remain the most accepted and widespread way to authenticate users. In this regard, users are experiencing an explosion of usernames and passwords making it difficult for them to remember all the credentials they have. At this point users have two options: use password managers or reuse the same passwords across different services. At best, password managers are used to store all their login data in a single hopefully-secure container. Should someone succeed to steal or access that container, the user is fully exposed. Nevertheless, the most widespread option is still to use the same low quality password (or small set of password), in multiple services, putting their online security at risk.

So far, the best partially successful identity federation systems available are online solutions, where users reuse their accounts at third-party websites to log into a service. This is convenient for users who do not have to establish separate accounts with separate passwords and are presented with a consistent interface for logging into services. Nevertheless, it is detrimental for security and privacy.

Traditional solutions introduce a single-point-of-failure in the system, since the IdP is involved in every authentication to a service provider. The IdP is able to impersonate its users if it acts maliciously. Furthermore, it may act as a “Big Brother” that can track the browsing behavior of its users and link their accounts across different services.

In response to the above problems, the European project OLYMPUS [6] (ObLivious Identity Management for Private and User-friendly Services) presents a way of managing identity in a privacy-preserving way, based on novel cryptographic approaches applied to currently deployed identity management technologies. OLYMPUS, in particular, uses distributed cryptographic technologies to split up the role of the online IdP among multiple authorities in a way that eliminates the single-point of failure and the ability to impersonate users from a malicious IdP. The proposed system prevents these problems and also establishes a strong relationship between real identity, digital identity and additional derived identities that make it possible to carry out transactions in a privacy-preserving way, while guaranteeing their legitimacy. OLYMPUS tries to facilitate integration into existing systems by minimizing hardware requirements and providing user-friendly authentication with a user and password, as usual.

In Internet of Things (IoT) scenarios, OLYMPUS also represents a promising approach. IoT devices are large consumers of services both inside and outside our home networks. Providing adequate protection by enabling privacy-preserving transactions to these types of devices is possible with OLYMPUS. However, the special requirements of IoT scenarios must be taken into account. For example, strong authentication of IoT devices is necessary to ensure reliability. Moreover, some IoT scenarios require offline Device-to-device (D2D) interactions without explicit user intervention. The offline privacy-preserving authentication approach also supported in OLYMPUS fits perfectly into these scenarios. OLYMPUS architecture leverages actual privacy-Attribute Based Credentials (p-ABC) [7] solutions, so that the p-ABC credential issuing process between the IdPs and the user is performed in a distributed way. Those credentials can then be stored for a mid-term period in the IoT device, which runs the privacy-ABC mechanism to present derived crypto-proofs to the verifier IoT device.

The OLYMPUS framework is under development and being validated and tested in two main use cases. The first one is related to online eCommerce scenarios that require strong authentication and high level of assurance and trust, whereas the second use case is intended to minimize the data that is revealed to a merchant when buying restricted goods and services using a mobile ID credential such as the mobile Driving Licence (mDL).

This paper presents the OLYMPUS ecosystem, including the main addressed requirements, the proposed architecture, an overview of the cryptographic building blocks, as well as the use cases involved. Also, the benefits of the OLYMPUS solution for strengthening security and trust in online and offline identity processes are discussed, particularly how user privacy is increased by using distributed cryptographic techniques to divide the role of online IdP among multiple authorities, so that no authority can impersonate or track users. This paper is an extended version of our previous conference paper [6], presenting the novel OLYMPUS identity management architecture, extending the related work, adding detailed description of its applicability in use cases, and including the security analysis of the proposed system.

The rest of this paper is structured as follows. Section 2 describes the state of the art in the identity management field, while Section 3 describes the OLYMPUS approach, introducing first the requirements of the project, then the proposed architecture and finally cryptography building blocks. Section 4 delves into the use cases for evaluation. Section 5 brings an overview in IoT scenarios. Section 6 introduces a security and privacy analysis. Finally, Section 7 closes the paper with the main findings and conclusions.

## 2. State of the Art and Background

Traditional identity management services (IdMs) rely on the usage of centralized identity providers (IdP) which create, maintain and manage information about the identity of its users while providing authentication services to relying parties (RPs). Those IdPs enable the use of Single Sign-On (SSO) technologies that allow users to only perform the authentication process once. However, *linkability issues* appear in these scenarios. Same personal information is stored by different providers making users traceable by, for example, linking user names and emails between services or comparing attributes that are specific enough. Existing identity management solutions try to address authentication scenarios with different approaches.

X509 [8], based on Public Key Infrastructures (PKI), only needs a minimum amount of trust for its deployment. The user trusts a certification authority (CA), accessible at the time of issuance, which will issue a digital certificate for a set of user attributes. When a user wishes to authenticate to a service through this system, the service must rely on this CA as a valid issuer and only the user and the service will be involved in the authentication process.

Although it is an easy system to implement, this approach has significant challenges. First, service providers must agree to use this system and must rely on one or more CAs as trusted entities. Second, users become an active part of security by being responsible for remembering and keeping their secrets safe (ideally there should be no user interaction, since deriving a key pair based on a password will allow for offline attacks given just the public certificate). It is a fact that users are not good at these tasks and most users have more than one device, which implies that private keys are copied along the different devices.

In Federated identity systems [9], also known as Single Sign-On (SSO), the trust requirement is reduced for each service provider by centralizing trust in a single identity provider (IdP). With this approach, the user only has to authenticate against (and provide information to) the IdP, eliminating the need of introducing the same data many times in multiple registering processes. Also, due to the centralized IdP-based solution, revocation is no longer an issue. Authentication against the IdP could be carried out in different ways, like certificate based solutions such as X509. However, this would lead to the same disadvantages mentioned before. To avoid this, the system can be based on the typical combination of username and password. In this case, the user is relieved of the responsibility of securely storing critical information and can easily login into a service using any device without having to make previous configurations.

In federated systems, IdPs can be in possession of authenticated attributes of the users like age, name or nationality. The authenticity of the attributes is assured by Attribute Providers (AP), which are organizations responsible for establishing and maintaining subject’s identity attributes. An example of AP could be a mobile driver’s license (mDL) issuer. The IdP can issue its own attributes or obtain user attributes from other APs (usually with user intervention).

With this in place, federated systems have the facility to provide a great level of granularity on authentication requests. The system allows the IdP to validate only the necessary attributes when making use of a service. For example, if a service wishes to verify that a user is above the age of 18, it is not necessary to leak the concrete birthday date. The IdP can now simply assert that the user is above 18 based on the info it has. This is a great way to ensure granular and selective disclosure.

Federated systems also have significant drawbacks. As in the case of X509, the linkability problem persists since user identification information is delivered to each service provider. Service providers are able to collect logs, identifying and possibly tracking users. Linkability problem is, thus, further aggravated with *traceability*. As a mandatory waypoint, the IdP learns which services users are accessing and an create a profile based on the history record. Furthermore, the IdP becomes a *single point of failure*. If the IdP manages user attributes and it is compromised, all personal information it contains may be affected or leaked.

Within federated identity systems, there are several possibilities for implementation. SAML and OAuth represent the two most widespread approaches. SAML is a business-centric approach. Defined by the IETF through an RFC [3], it is also an OASIS standard. Messages in SAML are XML and communication is carried out by combining SOAP and HTTP protocols. SAML is not a lightweight protocol, but it is relatively simple to implement. The protocol specifies the issuance of a token signed by an IdP. In this regard, the service provider must maintain the public key of the issuer in order to check the validity of the signed token and its time stamp. The main disadvantage of SAML is that anyone who owns the signed token can impersonate the user, so SAML tokens must have a very short validity time.

Similarly, OAuth [2] is a standard that leaves many aspects of deployment open. This makes it harder to implement and deploy. Unlike SAML, OAuth messages are based on JSON and transmitted directly via TLS. OAuth allows various workflows and different types of tokens to be used. For example, OpenID Connect [10] is an implementation of OAuth similar to SAML, which is based on the use of bearer tokens. These tokens have a limited validity in time and are verified by the signature made by the IdP. Another possibility is that the service provider contacts the IdP to perform the verification of the validity of the token. OAuth is implemented by federated identity providers such as Google, Facebook or Twitter.

These technologies try to provide security and privacy features in federated online scenarios. However, in these solutions the IdP becomes a single point of failure. Even more, if it acts maliciously, it would be able to impersonate its users. Also, being an element involved in all authentication processes, the IdP becomes a kind of *Big Brother* able to track its users.

In that sense, systems are evolving so that users regain control of their personal data, improving their privacy. New concepts such as self-sovereign identity or privacy by design are being introduced [11] with this objective.

**Figure 1:**
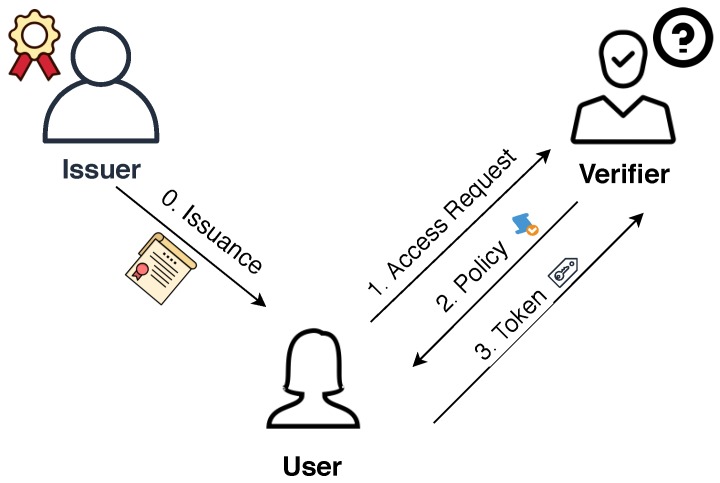
Privacy attribute-based credentials (p-ABC) flow.

Privacy Attribute-Based Credentials (P-ABCs) (Figure 1), are proposed as a possible solution to provide pseudo-anonymity, anonymity, and minimum disclosure of data. P-ABCs follow the same “offline” approach as X.509 where each user is provided with a digital certificate to securely access or use services. In P-ABC systems, like in X509, users get a credential from an issuer that contains a set of attributes which are asserted to belong to that specific user. However, the main difference with X509 certificates is that these P-ABC credentials allow the derivation of dedicated one-time tokens that only reveal the strictly necessary information.

In these scenarios, when the user wants to access an account or resources at a service provider (SP), the provider first responds with a presentation policy, stating the requirements the user has to fulfill. The requirements can be from simple re-authentication requests to requests for proofs of certain attributes like “is the user over 18 years old and lives in an European country?”

If the user’s credential can satisfy the policy, it derives a *presentation token* from the credential. A single presentation token can contain information from any number of credentials and can reveal a subset of the attribute values in the credentials.

P-ABC systems involve user centric approaches by giving the data control to the final users and enabling them to choose when, what, how and with whom to share their information. An ongoing problem in current authentication systems is the huge collection and processing of personal data. In that sense, the possibility of performing a selective disclosure represents a major improvement. P-ABCs grant a secure and trustworthy verification of individual attributes out of a larger credential without giving away the whole set of personal data. Moreover, the attributes available for selective disclosure are all those that can be verified by the issuer at the time a credential is issued.

P-ABC presentation tokens are, by default, unlinkable. This means that unless the disclosed attributes specify it, a receiver is not able to know if two presentation tokens come from the same or different users. Also, given two presentations tokens, the verifier cannot know if they were derived from the same credential or not. Giving users the ability to control linkability is one of the key concepts in P-ABCs.

In certain scenarios, such as when it is convenient to maintain a web session, having total unlinkability may not be desirable. In these cases, users can create tokens based on pseudonyms, which can be understood as a version of traditional public keys focused on preserving privacy. Pseudonyms are derived from the user secret contained in the credential in a way that preserves unlinkability between them, regardless of the number of pseudonyms derived from the same secret.

Although proposals such as Identity Mixer [7], U-Prove [12] or European projects such as ABC4Trust [13] and ARIES [14] represent good ideas for the deployment of P-ABC systems, their adoption is still very limited. Challenges with these systems, such as the difficulty of implementation or poor usability, is proving expansion to mainstream services difficult. P-ABC systems keep suffering from some problems similar to those already found in X509. For example, they require users to keep their credentials secure and to manage them correctly. In addition, mismanagement of credentials could allow attackers to impersonate the user. Solutions such as protecting cryptographic material with smart cards are useful but not always convenient as they require users to have card readers and applications on their devices. Moreover, the P-ABCs tokens verification or handling in comparison with traditional signatures and credentials is more complex. While traditional SSO systems use widely supported standard cryptography methods, in P-ABCs the underlying signature schemes, proofs and other mechanisms used require custom implementations.

Finally, another major problem in existing P-ABC schemes is their reliance on a single Identity Provider to issue attribute-based credentials. These scenarios leave issuers as single point of failures. If a given issuer acts maliciously, its possible to impersonate its users compromising the integrity and privacy of the system.

European project OLYMPUS [6] (ObLivious Identity and Management for Private and User-friendly Services) is devising a privacy-preserving identity management solution employing distributed cryptographic techniques to split up the role of the online IdP over multiple authorities, so that no single authority can impersonate or track its users. Moreover, by establishing solid links between citizens’ physical and digital identities, the derivation of privacy-preserving digital identities will enable better transactions backed by strong cryptographic mechanisms.

## 3. Olympus Approach

The OLYMPUS approach evolves traditional methods of federated identity management by eliminating the identity provider (IdP) as the single point of failure. The central idea is that IdP will no longer be able to track its users through its access to service providers (SPs). Advanced cryptographic protocols are used in OLYMPUS to distribute the work traditionally done by a single IdP among a set of *N* different IdPs where none of them needs to be totally reliable while maintaining the privacy and security guarantees of the system as long as all the IdPs of this set have not been compromised. The set of all *N* partial IdPs is called *virtual IdP* (vIdP).

This vIdP addresses all the tasks that were previously performed by a single IdP.

### 3.1. Requirements

With these considerations, OLYMPUS Identity Management system identifies the following requirements for its architecture, defined later on.

- **No Impersonation by IdPs**: A coalition of less than all IdPs in the OLYMPUS system will not be capable of impersonating the user or generating a token or credential. That is, a subset of malicious IdPs cannot issue access tokens for any Relying Party (RP). The architecture follows a distributed approach minimizing privacy and security risks.
- **Avoid offline attacks**: Brute forcing a password of a user can only be done if all servers are corrupted. Even the collection of n−1 stored account information’s $acc_{i}\left[uid\right]$ does not allow the attacker to perform an offline attack of the user password.
- **Short-lived authentication tokens**: User should employ short-lived access tokens after authenticating to the system. These tokens can last from just a few seconds up to several hours. However, after they expire, the token no longer allows any kind of action. In addition, mid-term tokens can be employed to support offline machine-to-machine (M2M) scenarios.
- **Unlinkablity across Relying Parties (RPs)**: The identity of the users cannot be linked between different RPs. Access tokens are unlinkable. An attacker will not gain knowledge by mere observation of the tokens because he will be unable to relate the items of interest of a user in a meaningful way.
- **Hide RPs from IdP**: IdPs will only be aware that an authentication process is taking place. They will know the user who is performing an authentication so they are able to assert the necessary data and attributes, but the IdP will not learn which relying party the user is contacting.
- **Minimize user-side hardware/software**: Reduce or avoid the storage of secret information on the user’s side as well as the execution of advanced code. In addition the system will not require that the user has any specific hardware. Nonetheless, the framework should also support offline scenarios, so the user might need to store mid-term material on his side.
- **Data-minimization**: The collection and processing of personal data must be minimized and adequate for carrying out the purpose for which the data is processed. The framework should provide selective disclosure of personal attributes, in different contexts.
- **Easy integration with existing IdM technologies**: It must be possible to integrate the solution with standards such as SAML and OAuth without implying major changes for the service providers.
- **Optional support of anonymous credential Systems**: It may be necessary to manage credentials that allow minimal disclosure, for example in offline use cases. Supporting the management of P-ABCs adds robustness to the approach.
- **Credentials might be linked to soft-proofs**: Including biometrics, location information, and contextual information of the mobile device.

### 3.2. OLYMPUS Architecture

**Figure 2:**
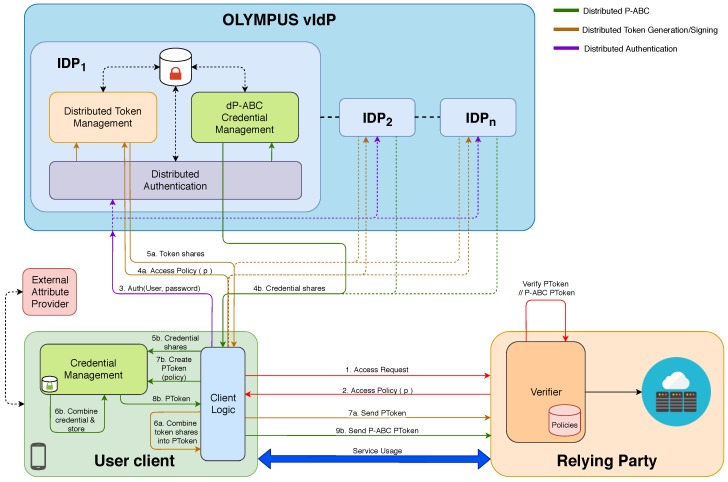
OLYMPUS architecture.

To address the requirements presented in Section 3.1, OLYMPUS proposes an architecture shown in Figure 2. On the figure, steps common to the distributed token and dP-ABC scenarios are numbered without a letter (1–3); steps exclusive to the distributed token scenario use a number followed by the letter *a* (4a–7a); and steps exclusive to the dP-ABC scenario use a number followed by the letter *b* (4b–9b).) that divides its operation into two well differentiated processes. (1) Authenticate the user, and (2) Issue an access token or credential, depending on the scenario, by the use of cryptographic solutions to distribute these tasks among a set of servers.

For user authentication, OLYMPUS architecture’s modular design enables the support of any authentication method with simple implementation changes. Nonetheless, the development of the project will be focused on authentication by username and password for several reasons. First, one of OLYMPUS goals is reducing trust requirements on user devices and this authentication method does not need any secure storage on user side. In addition, usability is improved because users do not need to configure any kind of certificate before authenticating in any device. What is more, usual security concerns about passwords are reduced because only one password has to be managed and distributed cryptographic techniques are used. Lastly, this method does not entirely rule out the use of secret keys or certificates for authentication. If a user must or wants to rely on them (for example, an IoT device without user input), he can use a secret to derive a username and password for the use on OLYMPUS. Considering the focus on username and password based authentication, and for the sake of simplicity, our solution will hereafter be described assuming this method is used.

OLYMPUS architecture supports two different approaches to deal with distributed authentication, token generation and access to services. On the one hand, in the *distributed token approach*, the user client sends the specific access policy to the IdPs, which perform a distributed threshold-based signature inspired by PASTA [15], described in Section 3.4.1. Then, the client receives the set of signed token fragments from the IdPs that he recomposes into the one-time access token. Once this is done, the client automatically presents this standardized access signed token to the Relying Party. The relying party is the service provider that requests some kind of authentication from the user to grant him access to the service, and deposits its trust in the OLYMPUS vIdP for this authentication.

On the other hand, in the *distributed P-ABC credential approach*, the client receives from the IdPs a set of credential fragments that he recomposes into a fully P-ABC credential. After that, the credential is stored locally in a secure way (e.g., mobile wallet), and the user can generate itself privacy-preserving crypto-tokens to be presented to the relying party. In this latter case, unlike the former, the same obtained credential can be employed several times to derive unlinkable tokens, and therefore, users do not need to be online to interact with the IdP to get the token (as in the first approach). Thus, it enables face-to-face scenarios where the verifier relying party can be accessible by the user through short-range wireless communications (e.g., Bluetooth, NFC, 802.11p), suitable for IoT, where the Verifier, or even the user, might be impersonated by a smart IoT device.

The Olympus architecture is complementary to other approaches for ensuring strong user authentication, such as multi-factor authentication. That is, the focus of Olympus is on increasing security of password authentication through distributing trust only. However, Olympus is compatible to standard approaches to multi-factor authentication. Given that the partial IdPs only issue a distributed token or credential once a user has proven to them that he/she knows its password, the user could in a similar manner prove that it holds the device it used during registration (a common approach to achieve a second factor). This can be done using standard methods in a non-distributed way, but it is also possible to get distributed security for this second factor. Consider for example the case where the second factor is an authentication/code app where a One-Time Password (OTP) is constructed to a specific user based on a user-specific seed and a time stamp. If instead of having a single seed we have a seed for each of the partial IdPs, then the OTP from the app would be a combination of an OTP based on each of the partial IdPs’ seed. The partial IdPs can then exchange among each other their partial OTPs of that user for that specific point in time in order to verify correctness.

The compatibility of OLYMPUS with standards such as OAuth or SAML is given by the ability to serialize the distributed tokens in messages compliant with these protocols. For example, in the case of OAuth, tokens are serialized using the JSON Web Token (JWT) standard which is the representation used by this protocol. The token is verified in the same way as a common JWT token by adding the underlying cryptography to support the OLYMPUS framework.

As can be seen in Figure 2, the OLYMPUS architecture describes three main roles: the user, the virtual Identity Provider (vIdP) and the Relying Party (RP), which are describe in the following sub-sections.

#### 3.2.1. Virtual Identity Provider (vIdP)

The virtual identity provider is the core concept of the architecture. OLYMPUS considers several identity providers (IdPs) which do not have to be fully trusted. Each of the IdPs has three specific modules: one for authentication and two for the issuance.

*Distributed Authentication*: It is in charge of checking the provided user name and password based on secret distribution protocols [16]. It works together with the rest of the authentication modules and once the validity has been checked, the user is authenticated in the OLYMPUS vIdP.

*Issuance*: the distributed issuance can be done in two different ways either using tokens or ABC credentials:

- *Distributed Token Management*: It is in charge of distributed token generation. Each IdP generates a token share given an access policy. When all the necessary token shares have been generated, the user client will be able to combine them into a valid access token to be presented to the relying party.
- *dP-ABC Credential Management*: It is responsible for the management of distributed credentials. As in the distributed tokens approach, each of the IdPs will generate a share (credential share). However, unlike the former, which generates shares related to an access policy, the credential generation will contain all the user attributes. Once all the shares have been generated, the user will be able to combine them in a full credential, which, unlike in the previous approach, might be used several times to derive crypto-proofs to be presented to the relying party.

#### 3.2.2. User Client

The user client is involved in the authentication process, sending the username and password to the vIdP. In addition, it supports different components to deal with the distributed authentication and the two privacy-preserving approaches introduced above, that is, distributed token generation and distributed P-ABC. These functionalities are carried out by two different modules:

*Client Logic*: It deals with the interaction between the service the user wants to access and OLYMPUS.

- *Authentication*: It is responsible for performing the distributed user authentication and then selecting the access method to be used that is, tokens or P-ABCs.
- *Token handling*: In the case of tokens, there is a sub-component in charge of transmitting the access policy of the service to the vIdP and then combining the token shares received in a valid access token. In the case of distributed P-ABCs, there is a sub-component that allows obtaining credential shares from the OLYMPUS vIdP and entrusts the composition of the credential and its storage to the credential management module. When the client wants to use the credential, the access policy is communicated to the credential management module, generating an access token.

*Credential Management*: this component is employed in the p-ABC setting, for the offline case, and it is in charge of composing the complete p-ABC credential with the credential shares obtained from the OLYMPUS vIdP. In addition, the credential is securely stored in an internal wallet for a short amount of time and only in offline cases. The module is able to generate access tokens of the stored credential for a given access policy.

#### 3.2.3. Relying Party (RP)

The relying party protects access to a range of resources or services. Its main purpose is to generate the access policies that the user will need to satisfy to access to the service. The user will need to agree the policy and give consent for revealing the attributes. Consent handling and access policy generation are out of scope of the OLYMPUS framework, and different consent and policy mechanisms can be on-boarded. The framework is presented with a policy and will generate a satisfying proof, while the consent will be handled by an external client application. The RP plays the role of Verifier, so that it needs to validate the signed access tokens presented by the user. Thus, two main components are involved.

- *Verifier*: It is responsible for verifying the validity of submitted access tokens (including checking the signatures) and whether or not these tokens are fitting for a given access policy. The Verifier can implement any of the approaches introduced above, that is, verification of the distributed tokens integrated as OAuth or SAML assertions, or verification of distributed p-ABC crypto tokens derived from the user credentials.
- *Policy DB*: It contains a set of policies defined for a particular service or services, that is, specific user attributes, their format as well as the predicates related to the attributes. Access policies may be described via XML or JSON.

### 3.3. Main Architectural Identity Management Processes

In OLYMPUS, there are two phases. The registration/enrolment phase and the generation phase, in which a registered user can obtain tokens or credentials.

#### 3.3.1. Registration Phase

It allows a user *U* to create a fresh user account with the distributed identity provider (vIdP). The account is created for a user name and protected through a password *pwd*. The registration is an interactive phase between *U* and all identity providers that compose the vIdP. The enrolment is performed using a user name, a password, and optionally a set of attributes *A*. These attributes would have been obtained by the user from an external Attribute Provider (AP), so the vIdP can verify their validity before accepting and storing them. At the end of the registration protocol the user receives a confirmation message indicating the successful completion of the account creation. From this moment, the user has an account in the OLYMPUS vIdP.

#### 3.3.2. Generation Phase

The generation phase begins when a user *U* wants to make use of a service *S* offered by an RP (steps *1* and *2* of the Figure 2). This process is performed out-of-band of the OLYMPUS framework and different mechanisms to handling the policy agreements can be on-boarded (such as the one implemented in ABC4Trust project). The main objective of the generation phase is to perform authentication and token generation/presentation in a distributed way.

##### Distributed Authentication and Token Generation/Presentation

The authentication process begins when *U* provides its username *user* and password *pwd* to the vIdP (step *3*). Upon successful authentication towards the identity providers, there are now two possible scenarios. (1) Distributed token generation/signing (for SSO) and (2) distributed P-ABC, mainly for offline scenarios.

1. *Distributed Token Generation/Signing* In the SSO case (1), *U* gets a valid access token for the *P* access policy required by *S*. The token is generated in a distributed way through the OLYMPUS IdPs infrastructure (steps *4a, 5a* and *6a*). This distributed signing process is built on the grounds of a *threshold signature scheme* and a *threshold oblivious pseudorandom function* (TOPRF), as explained in Section 3.4.1. Then, *U* presents this token to *S* (step *7a*) and, after verifying it, *S* makes the decision to grant the corresponding privileges.
2. *Distributed P-ABC*: in the P-ABC case (2), unlike in previous one, *U* does not get an access token. He gets a P-ABC credential that has been generated in a distributed way (steps *4b, 5b* and *6b*), like the access token in (1), by the OLYMPUS IdPs. Now *U* is able to use a service *S* that requires an access policy *P* by deriving himself an access token based on the previously obtained credential (steps *7b* and *8b*). *U* can hold the credential securely in his device, opening up the possibility of an *offline* case where the user can generate the access token without contacting the vIdP (thus, jumping directly from step *2* to step *7b*). This scenario is especially useful when the user foresees that he will not have connection to the OLYMPUS infrastructure at the time of making use of a service. For instance in IoT use cases where the IoT device needs to interact directly with another IoT device and there is no internet connectivity to get short-live tokens. Credentials issued with a certain lifetime can cover this scenario, with the minor inconvenience of having to manage them on the user side.

### 3.4. OLYMPUS Building Blocks

#### 3.4.1. Building Blocks for the Distributed Authentication and Token Generation/Signing Approach

The OLYMPUS framework takes as a starting point the notion of *threshold cryptography* [6], a type of cryptography where secrets and other cryptographic material are shared between several parties. The sharing works in such a way that knowing less than all shares does not give any information away about the secret. It is this idea that makes it possible to achieve enhanced security by using a set of *n* IdPs rather than a single one. However, this in itself is not enough, as for example sharing a hashed password in a threshold scheme will still not allow the IdPs to verify said password without it existing in plain in their memory at some point in time. Fortunately, several techniques exist to achieve this, which we leverage in the OLYMPUS framework [15,17]. In particular, we take significant inspiration from the PASTA framework [15], which is composed of a threshold signature scheme and a threshold oblivious pseudorandom function (TOPRF).

In a threshold signature scheme, the private signing key is split into a number of partial keys and shared among the *n* IdPs [6]. This allows each IdP to compute a signature using their partial key, and because of the mathematical structure in certain popular signature schemes such as RSA [18] or (EC)DSA [19], a user who knows all the partial signatures can combine these into a complete signature. Thus the signature resulting from the combination, is a standard signature and can be verified by parties who are not aware that it was constructed based on a threshold signature scheme. A bit more formally we consider the setting where IdP *i* holds private signing key share $k_{i}$ of the combined key *k* and can compute a partial signature on the message *m* as $Sign_{k_{i}}\left(m\right)=x_{i}$. All of these can then be combined $Combine(x_{1},\ldots ,x_{n})=x$ in a signature *x* which can be verified using a public key *p* (corresponding to the private key *k*) by computing $Verify_{p}\left(x,m\right)=$ ACCEPT if and only if *x* was a valid signature on *m*.

The TOPRF implements the idea of a pseudorandom function, PRF, shared between the *n* IdPs so that they must work together to compute PRF(m)=y on message *m* [6]. However, this must also be done obliviously, meaning that none of the IdPs are allowed to learn *m* or *y*. Like the case for threshold signatures, each of the IdPs will get a share of a private key; $k_{1},\ldots ,k_{n}$. The user who wishes to query the TOPRF on *m* will first encode this using some randomness ρ. That is by computing $Enc_{\rho }\left(m\right)=x$. The user then sends *x* to the servers who compute their shares of the PRF output; $Eval_{k_{i}}\left(x\right)=y_{i}$. They send this back to the user who can then combine this into the PRF output; $Combine(y_{1},\ldots ,y_{n})=y$.

Based on these components the overall PASTA scheme can be described as follows [6]:

**Setup** The IdPs setup a threshold signing scheme among themselves and publish the public verification key *p*.
**Sign-up** The user constructs private keys for a TOPRF and distributes these to the IdPs along with an encoding of its password, pwd, using the TOPRF.
**Request** When the user wishes to get a token *m* signed by the identity provider, it picks some fresh randomness ρ and computes $Enc_{\rho }\left(pwd\right)=x$ and sends this, along with *m*, to the IdPs. Based on this, the IdPs construct an encrypted share of their partial signatures such that only the party who supplied the key shares for the TOPRF, and possessing the password, will be able to decrypt. The IdPs send their encrypted shares of the partial signatures to the user, who decrypts them, reconstructs the token signature and passes this on to the relying party, who can then verify it based on the identity provider’s public key.

Unfortunately, the PASTA framework does not give unlinkability between tokens issued to the same party [6]. The first step in fixing this is to randomize the token to be signed in some way unknown to the IdPs but known to the user, such that the user can remove this randomness before sending the token and its signature to the relying party. This can be achieved using something known as blind signatures [20]. It consists in the user picking some randomness and randomizing the message that the identity provider should sign. Because of a specific structure in the randomness, the user can remove it from the token while keeping the signature valid, before sending the token to the relying party. Such random signatures can be implemented on top of standard signature schemes such as RSA. Formally we can express this as $Blind\left(m\right)=m^{\prime }$ and $Unblind(x^{\prime })=x$ where $x^{\prime }=Combine\left(x_{1}^{\prime },\ldots ,x_{n}^{\prime }\right)$ with $x_{i}^{\prime }=Sign_{k_{i}}\left(m^{\prime }\right)$ for i=1,…,n.

A problem with blind signatures is that the identity provider now obliviously signs whatever a user gives it. This is an issue if the user is malicious [6]. For example, it could make the identity provider sign a token saying the user is older than he or she actually is. To prevent this, another cryptographic tool, known as zero-knowledge proof, comes into play. A zero-knowledge proof allows a party to prove that it knows something, called a witness, without leaking any non-public information about what it knows. This means that, based on some public information, it is possible for a party to prove that it knows some information which is hard to compute. Concretely, this could be that the public information is a SHA- 256 digest and the witness is a pre-image of it. The public information could also be an element of a large prime group and the witness the discrete logarithm of this element.

The aforementioned building blocks will be used to realize part of the functionality carried out by the elements of the OLYMPUS architecture (Figure 2).

Oblivious PRF will be used, as was the case in PASTA, in the process of distributed user authentication (namely in password verification). In particular, they will be a pivotal component of both the *Distributed Authentication* module of each IdP and the part of the *Client Logic* in charge of authentication. In the first case, the module will need functionality for the setup of the keyed PRF and the computation of the PRF output shares, while in the latter the main feature will be the combination of the shares to obtain the final output.

As for distributed and blind signatures (with the corresponding zero-knowledge proofs), they will be used for token generation in the first described case, that is, the single sign on scenario. The related functionality will be found on the *Distributed Token Management* component of the IdPs, the part dedicated to SSO on the user’s *Client Logic* and the *Verifier* of the relying party. The IdPs will have to deal with key management and computation of signature shares requested by the user, while the client logic will have to combine the shares in a presentation token to send it to the relying party, which will verify its validity.

For further reading on the mathematical description of the cryptographic techniques that are being developed for authentication and token generation in OLYMPUS refer to Reference [21].

#### 3.4.2. Building Blocks for the Distributed p-ABC Approach

On the other hand, OLYMPUS will support applications where the user has to authenticate and present some specific attributes while preserving privacy, selective and minimal disclosure of personal information. For this purpose, it will rely on P-ABCs, an idea proposed by Chaum [22,23]. The IdPs will issue in a distributed way a P-ABC credential that contains all of the user’s attributes. The user can then derive the final presentation token using zero-knowledge crypto proofs, so he can selectively disclose a set of attributes. These credentials can be stored, enabling an offline use case where the user derives a presentation token for a relying party without contacting the IdPs. In fact, the user could carry out minimal disclosure proofs for different policies (for the same or a different relying party) using the stored credential.

It should be noticed that the offline scenario could be realized not only with a distributed p-ABC solution, but also with the approach based on distributed blind signatures described in the previous section, as long as the tokens are given a longer life and kept in the user side. However, unlike the p-ABC approach, that method binds the token/proof to a certain Relying Party acting as verifier and known at time of issuance. Giving that usage of those tokens against different verifiers is not possible, and Relying Parties may not be not be known beforehand at token issuing time, this scenario has practical limitations.

In addition, our proposal ensures that ABC credentials are mid-lived (for example, valid only for a few hours), so the corruption of a user device will not result in a permanent compromise of his identity data. Even if the attacker learns the attributes contained in the credential, he would not learn later changes or additions. What is more, even if there is no change on the information, once the credential expires the attacker will not be able to generate any proof for the user’s attributes, so there is a short window for possible malicious operations. This is an important feature because a crucial design goal of OLYMPUS is to avoid any trust assumptions for the clients’ devices.

There exists several realizations of P-ABCs in the literature, such as the strong-RSA based and pairing-based schemes by Camenisch and Lysyanskaya (CL) [24,25] or Pointcheval and Sanders (PS) signatures [26]. A credential scheme that could be appropriate for OLYMPUS is the one presented in Coconut [27]. In this scheme, the issuance protocol follows a threshold strategy, where *t* out of *n* partial credentials can be aggregated to obtain a consolidated credential. These credentials are unforgeable, that is, it is impossible to obtain a valid one using fewer than *t* partial credentials (for example, in the case of a coalition of fewer than *t* malicious issuing authorities). In addition, the scheme fulfils security properties such as hiding of private attributes (both from issuing authorities and verifiers) and unlinkability of multiple selective attribute revelations. However, security against transient corruptions, often called proactive security like in References [28,29], cannot be accomplished with this scheme. The idea behind this kind of security is periodically refreshing the secret shares held by the parties without changing the underlying secret. That way, even if an adversary accomplishes the corruption of some shares, as long as it does not learn the whole secret (in a threshold scheme, learning at least *t* shares) it will have to start from scratch on the next period. As one of OLYMPUS goals is obtaining a proactively secure identity provider, it will have to deviate from this scheme, developing a P-ABC system that satisfies its specific needs.

Distributed P-ABCs are handled on several elements of the OLYMPUS architecture presented in Figure 2. The *dP-ABC Credential Management* module on the IdPs needs to setup and manage the necessary cryptographic material for the scheme as well as computing the credential shares for each user. On the user’s side, the *Client Logic* takes care of the execution flow and the communication with the IdPs and the relying party, while the *Credential Management* module needs to provide the functionality to combine credential shares, securely store the user’s credentials and derive presentation tokens suited for specific access policies. Lastly, the *Verifier* on the relying party needs the logic to check if a presentation token conforms to a specific policy and was derived from a valid credential.

For further reading on the mathematical description of the cryptographic techniques that are being developed for distributed P-ABCs in OLYMPUS refer to Reference [30].

## 4. Use Cases

This section introduces the use cases, currently under development, that aim to validate the usability, performance and viability of the proposed architecture, including distributed approaches to authentication and credential and/or token issuance. In this sense, the *Credit file* use case, aims to validate the novel OLYMPUS approach for distributed signing/generation of one-time threshold-based tokens, whereas the *Mobile Driver License* use case, is intended to showcase the offline approach using distributed p-ABC credentials.

### 4.1. Use Case: Credit File

**Figure 3:**
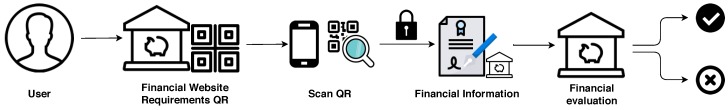
High level overview of Credit File use case.

Figure 3 shows a high level overview of the Credit File use case. A user wants to form some kind of contractual relationship with a financial entity, which generates a QR code that contains the requirements that the user must fulfill. Then the user can scan the QR code and use that data to generate (if he desires so) a message that contains exclusively the needed information with proof of its authenticity. This would be the step where OLYMPUS brings its value. The financial entity would then use the information presented by the user to evaluate the proposal, accepting or rejecting it.

The main objective of this use case is to change the paradigm that describes how the relationships between financial entities and potential customers are performed from a perspective where the amount of information exchanged between both of them is minimized.

In order to achieve this goal, the idea is to build an online platform aimed at SMEs, self-employed and legal or natural individuals whose purpose is to create and manage their financial report for being used during their interactions with the entities. The motivation for this approach comes from the fact that, currently, for a customer to get some kind of financing he is required to provide a great amount of personal information before knowing if the actual financing is going to be granted. In addition to that, as per the EU General Data Protection regulation, it is required for the potential customer to sign one or more documents giving consent to provide this kind of personal data and for the entity to keep both the personal data and the consents during several years. Thus, within this use case we aim to ease this interaction allowing the potential customer to exchange the minimal required information until the request is granted.

The goal of this use case is to provide the entity with an anonymous financial report with only the minimal information required. This information allows the entity to evaluate if the user is suitable for a specific product or service. Moreover, performing the interaction in an anonymous way guarantees that the entity’s decision is not influenced by any personal details about the user, being based solely in objective data. Hence, the entity can not discriminate any potential customer on any non-relevant information. This is an added advantage to the immediate ones of promoting the user’s privacy and reducing the bank’s need to process sensitive information. Once the evaluation of the credit file’s information is performed, the bank produces a response and if the user wants to accept the contractual relationship, he or she will be able to reveal his or her real identity.

In this context, the bank assumes the Relying Party role in the architecture, while Credit File acts as an attribute provider for the client, which would be the OLYMPUS the user.

**Figure 4:**
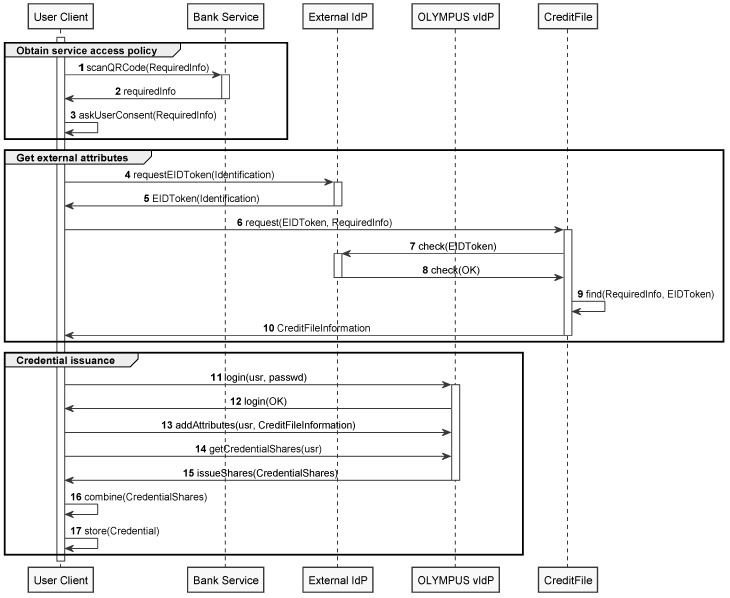
Credit File issuance process with OLYMPUS.

The process begins with the potential customer using the Bank Service website to select a profile that determines the required information to be provided (the required information) and, therefore, the type of request that is going to be performed. Once the user evaluates these information requirements, a QR code will be generated containing a ciphered description of the data to be retrieved from the financial report (Figure 4, steps 1–3).

Then, on a second step, the user starts the collection of the necessary information. Credit information can only be obtained through fully authenticated requests and for this purpose, the user will use an external identity provider to prove that he is who claims to be (steps 4–5). With the identification obtained, the user goes to the Credit File platform, which is a financial attribute provider, to obtain a financial report on behalf of the user (steps 6–10).

At this point and following the processes defined in Section 3.2, the user, through OLYMPUS, will be able to obtain the necessary material to anonymize the process. First, the user logs-in with his username and password in the OLYMPUS vIdP (steps 11–12) and then, the user adds the obtained creditFile to his attribute collection (step 13). Once this is done, the user is able to request the issuance of a credential containing all its known attributes, to the OLYMPUS framework, or a token that proves the possession of a certain set of attributes. The above example shows the offline case, so a credential will be generated and stored by the user in his device and can be used to generate P-ABC proofs (steps 14–17). For the online setting using blind signatures, the client sends to the IdP the policy to be fulfilled, and then, the IdPs that form the vIdP, generate the shares of a token that the client can compose into a valid token to present to the service.

**Figure 5:**
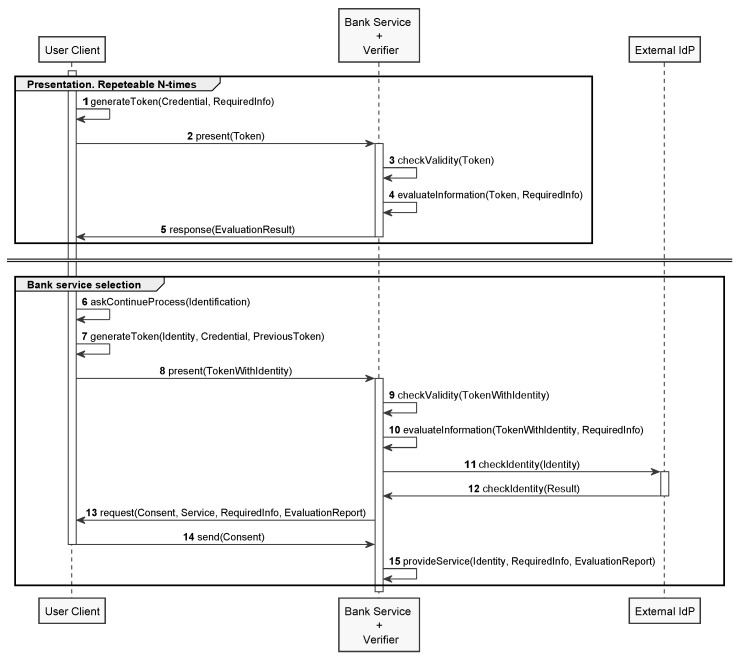
Credit File presentation process with OLYMPUS.

With the issuance process completed, the user has a P-ABC credential with all his attributes. Now, with the policy specified by the banking service, the user can construct an anonymous proof with the minimum necessary information and present it to the service (Figure 5, steps 1–2). The banking service receives the user’s test and proceeds to the validation of this proof as well as to the evaluation of the attributes contained according to its own policies. This process ends with the issuance of an evaluation result (steps 3–5). The user can repeat the generation and presentation of proofs in multiple banking services until he finds the one he likes, thanks to the possession of his complete credential.

Finally, once the user chooses the banking service, it will be necessary to identify himself and cease to be anonymous. In this sense, the user will be able to create a new proof that contains his real identity and the previous proof to present it again (steps 6–8). The banking service will then re-verify the presented proof as well as the information contained and additionally the identity of the user (steps 9–12).

Finally, the banking service will make a request for explicit consent from the identified user, for the service required and with the evaluation report obtained. In this way, when the user accepts, a strong contractual relationship will be established between the user and the banking service (steps 13–15).

### 4.2. Use Case: Mobile Driver License (mDL)

**Figure 6:**
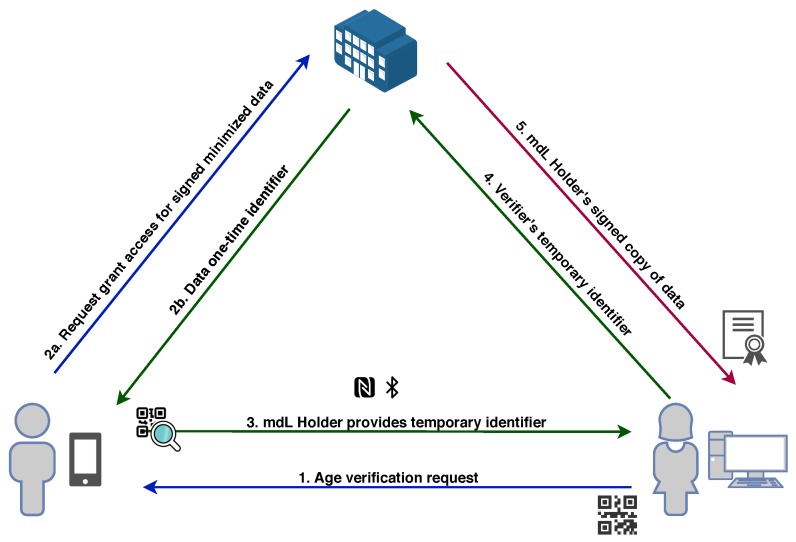
Mobile Driver’s License Use Case Triangle.

Mobile Driver’s License approach is described in Figure 6. The key aim of the proposed use case is to provide a new in person privacy-preserving verification scheme using smartphones only and no electronic or paper cards, ids, passports or other physical security documents. This serves as an alternative to the physical document and it is another way to verify oneself using just the mobile. In the context of this use case, a key feature to be achieved is *data minimization*, enabling partial personal information sharing at the absolute minimum required by a verifier. To achieve that, in a common interoperable manner, a new international standard named ISO 18013 (Driver’s License) part 5 [31] is being currently finalized and expected to be available within 2020.

This new specification will standardize an alternative form factor for a DL in mobiles. It will be particularly important as it is the first universal step towards a compliant common notion of personal id that can be stored in a smartphone (or other smart wearable), with broad adoption by the IT and software industry as well as authorities worldwide. The mobile DL will be available to connect and share information with the verifier’s reader upon approach. The mDL holder will be able to express his sharing consent [32]. Moreover, additional network interfaces are to be standardized as a next step – for instance, the WiFi Aware point to point access protocol.

In this use case, we show the process of age control when a citizen is willing to acquire some age restricted good or service (e.g., a purchase in a liquor store). In this situation, the mobile Driver’s License (mDL) app serves as the electronic equivalent to the physical card ID document. The main advantage in this case backed with our proposed approach is to support privacy protection through extended data minimization. The mDL includes functionality to allow showing the requested minimum amount of data appropriate for age verification and proving holder’s identity such as that he or she is older than a certain age (i.e., over 21), without effectively revealing the date of birth.

Based on ISO 18013-5 early committee draft [31], our main objective is to support the interconnection in the following two encounters:

1. The holder of the mDL contained in his smartphone and any mDL reader that needs to verify that (e.g., merchant verifier), and
2. The mDL verifier that needs to collect data about the mDL holder and receive it from the respective holder’s Issuing Authority (IA).

The approach is primarily structured to support in person verification, which is defined as *attended cases* in the standard ISO 18013-5. In the attended cases, any mDL verification shall be performed by a verifier person or entity. Remote verification is not supported yet.

Further applications of in person verification for individuals includes casinos, bars, entertainment theatres, social benefits and subventions, and anywhere else where access to services and/or products is limited to individuals above or below a certain age.

The estimated average usage per holder of an mDL is expected to be up to five times per week, just for age verification purposes. On top this, there are further ID verifications for picking up parcels, drugs from a pharmacy, and so forth. On the other side, a verifier will perform one check per transaction, totalling several hundreds per day. The verification is envisioned to be merged together with the payment process and that is why the proposed approach supports multiple channels of communication.

The mDL use case is enabling active privacy protection for face to face ID verification. As a result, the proposed approach tries to become the secure enabler of a data protection by design for the individual enforcing laws for data minimization such as EU GDPR [33]. Our approach tackles the issues of protection of anonymity of the user by enforcing the proposed distributed architecture.

In particular, distributed credentials proposed by the OLYMPUS approach (defined in Section 3.2) are particularly useful for the mDL use case. This technology provides the ability to minimize the attribute disclosure while maintaining user control over their data.

In the example where a user wants to buy in a liquor store, assuming he is registered in the OLYMPUS vIdP, the process is as follows. First, the user asks for the liquor and then the merchant asks for age verification to sell it (access request and access policy defined in steps 1–2 of the architecture Section 3.2). Now, by pairing his mDL app with the merchants verifier, the user obtains the access policy. At this point, if the user already has a valid credential issued by OLYMPUS and if he consents disclosure, the mDL app generates a proof for the given access policy (as long age requirement is met) containing, for example, that he is over 18 years old. If the user does not have a credential or the credential has expired, he must obtain a new one as indicated in the architecture Section 3.2 before generating the proof. At the end, the verifier checks the authenticity and integrity of the data and the good may be sold.

## 5. OLYMPUS Applicability in IoT Scenarios

IoT scenarios are comprised of smart devices, equipped with constrained hardware capabilities, which usually perform their processes without user attendance. Therefore, traditional password-based systems do not fit well in IoT. Instead, technologies based on X.509 certificates, Trusted Platform Modules (TPM) and/or shared key cryptographic systems are widely used to secure this type of IoT scenarios.

OLYMPUS architecture presented herein and its associated cryptographic technologies are also suitable for IoT scenarios, as the underlying cryptography being developed in OLYMPUS for the distributed P-ABC approach, requires less computational complexity with respect to the traditional P-ABC systems (e.g., based on CL signatures [25]). This fits perfectly with the computational restrictions raised from IoT scenarios.

Smart devices may need to access third-party services that require the authentication of these devices, an essential step that must occur directly between the IoT device and its Identity Manager without end-user intervention applying the OLYMPUS approach. In addition, the support given by OLYMPUS to offline Device-to-Device scenarios through the use of distributed P-ABCs is also valuable for IoT scenarios. The proposed privacy-preserving mechanism for IoT transactions requires two main processes:

- *Bootstrapping and registration/discovery*: Performed against an identity manager or key manager in the IoT device home network, where the device authenticates itself (using, for instance, a pre-set certificate from the vendor) and retrieves the necessary crypto-material to be kept securely protected in the IoT device. There are proposals such as ARMY [34] that have already addressed this in a privacy-aware way. While this bootstrapping in the home network is outside the scope of OLYMPUS, the authentication through OLYMPUS is applicable. In contrast to other cases discussed in this paper, it will be necessary to manage and reuse the cryptographic material obtained during the first phase, replacing the role of the password as it is not appropriated for an unattended IoT device.
- *Authentication of the IoT device in an external service through OLYMPUS*: Once the IoT device finishes the Bootstrapping/registration/discovery process, the device gets cryptographic material with which it can start the next steps through OLYMPUS in order to use a third party service. For this authentication and the token handling, the Olympus setting based on distributed P-ABC credentials fits perfectly. The IoT device can obtain from diverse IdPs the p-ABC credentials and store them locally securely protected in an internal wallet. Then, during the presentation process against the target accessed IoT device or service, the calling device can derive Zero Knowledge Poofs from the credential to get access in a privacy-preserving way, ensuring minimal disclosure of information. Optionally, if the devices are extremely constrained, an IoT controller acting on behalf of their devices could take care of these credentials. Unlike other settings that uses p-ABC for IoT [35,36], our distributed p-ABC setting that is currently being implemented aims to reduce the computational complexity required for generating and validating the crypto-proofs, thereby making them suitable for IoT constrained scenarios.

**Figure 7:**
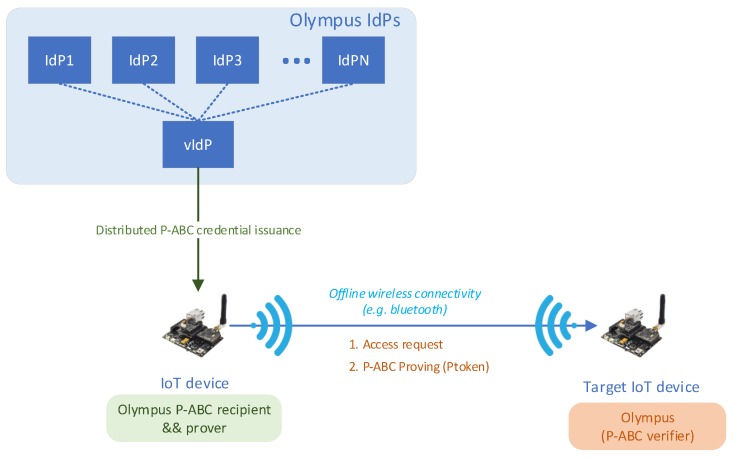
Olympus in the Internet of Things (IoT) setting for privacy-preserving M2M interactions.

Figure 7 shows a scenario where the OLYMPUS approach is applied to an IoT setting. An IoT device previously registered can authenticate to the OLYMPUS vIdP and request an ABC credential with a relatively long life time. Some time later, the IoT device may want to contact another device in an *offline* way, for example using Bluetooth or NFC. Then, the device would send an access request (step 1 in the figure), but the target device (that acts as the Relying Party of the OLYMPUS architecture) will grant access only if the requester fulfils some policy, such as for instance, attributes related to the manufacturer or vendor, device’s characteristics or attributes related to the IoT device’s owner. The IoT device can then use the stored p-ABC credential to derive ZKPs proving that it meets those requirements (step 2 in the figure), while keeping minimal disclosure and without contacting again the vIdP, so the whole interaction only involves *offline* communication.

## 6. Security and Privacy Analysis

### 6.1. Threat Model

OLYMPUS considers a distributed security model, meaning that all partial IdPs (forming a vIdP) must be corrupted in order for an adversary to compromise the security of the system. Concretely, we consider a corruption to be the setting where an adversary not only gains access to the data on a partial IdP, but is also able to control this partial IdP. The OLYMPUS system ensures that as long as only a single partial IdP is not corrupted, it is not possible to impersonate *any* user. Furthermore, it is not even possible to do an offline brute-force attack on the users’ passwords. This is so since the TOPRF used in distributed password authentication employs high-entropy randomness from each of the partial IdPs to ensure that the password-dependent information stored per user requires either the partial IdP-specific cryptography information from *all* partial IdPs or the user password *and* interaction between *all* partial IdPs. This prevents offline attacks, but still allows online attacks (where an adversary is impersonating a user). However, since the password authentication protocol of OLYMPUS ensures that all servers learn whether an authentication attempt was successful or not, they can throttle such requests to prevent password brute-forcing.

We note that the threat model does allow one more possible attack: If the adversary manages to corrupt a specific user, he/she is allowed to use whatever long-term token or offline credential the user might have stored. Thus the adversary will be able to impersonate this specific user for the lifetime of the specific credential/token. We do note however, that the adversary is not able to do so indefinitely as this would require to authenticate as the given user towards the vIdP in order to obtain a new credential/token. This is only possible if the adversary knows the user’s password.

Besides being secure against corruption of all but one partial IdP, OLYMPUS also supports proactive security. This means that if a partial IdP has been corrupted and then becomes uncorrupted, it is possible to refresh its secret material. Hence, this partial IdP will from then on be considered completely honest again and the adversary will not be able to use its previous secret information to impersonate it.

#### Privacy

One of the main privacy goals of OLYMPUS is to hide the users’ attributes from the RP, except the predicates on it reflecting the strictly required information for each specific user’s sign on request. For example an RP might learn that the user signing in is older than 18, but not the exact age of the person. However, all the user attributes will be known by each of the partial IdPs.

Another goal is to ensure unlinkability and untraceability. This means that the vIdP does not learn which RP it approves which policy for; and in fact it does not even learn the identity of any of the RPs the user accesses. In case of P-ABCs, the vIdP does not even learn the policies nor time which the users generate tokens for; however these two pieces of information are leaked to the vIdP when using distributed tokens.

Finally, we note that a user’s password will *never* be known to any party besides the user itself. Even if all partial IdPs of the vIdP get corrupted and work together, they will not learn the users’ passwords. If all partial IdPs collaborate, they will be able to launch a brute-force attack on the password for specific users. Hence, the worst case scenario of OLYMPUS corresponds to the standard scenario of a leaked database in a traditional hashed password verification scheme.

### 6.2. Analysis

We will not go into the details of the security model, nor the cryptography used. However, we note that the main approach to distributed password authentication and distributed token generation presented is formally proven secure using the UC-security model for cryptography and is presented in the work PESTO by Baum *et al.* [21]. The construction of a distributed signature scheme that is credential-friendly is given by Camenisch *et al.* [30]. Certain aspects and compositions have not been fully formalized and proven yet, in particular a fully fledged distributed credential scheme composed with the distributed password authentication protocol in PESTO, along with a distributed token management scheme using blind signatures and zero-knowledge proofs. However, we note that the latter is trivially possible to achieve since PESTO is secure under composition and composable secure blind signatures exists [37], as does composable zero-knowledge proofs of any statement [38].

## 7. Conclusions

This paper has presented the first distributed and oblivious Identity Management architecture that supports both, one-time unconscious authentication and access token generation/signing using threshold cryptography, and distributed p-ABC credential management to address offline scenarios. It has introduced an analysis of the security and privacy it can bring and the identified risks it faces. In addition, two use cases have been introduced and detailed how OLYMPUS can improve the privacy and security outlook in these scenarios.

The OLYMPUS architecture features key capabilities needed to achieve truly privacy-preserving Identity Management solutions, including unlinkability across both SPs and IdPs, no impersonation by IdPs, selective and minimal disclosure of private personal information as well as oblivious distributed IdP deployment that avoids a single point of failure. OLYMPUS is also beneficial for IoT scenarios, since its architecture and its efficiently developed cryptography techniques are suitable to address D2D offline IoT scenarios, comprised of potentially constrained IoT devices.

As future work, to cope with the current privacy-preserving identity management challenges in DLT scenarios [39], we envisage to apply the distributed p-ABC approach devised in Olympus to derive efficient and oblivious non-interactive crypto-proofs for blockchain, thereby achieving privacy-preserving data provenance in distributed ledgers.

## References

[1] De Clercq J. (2002). Single sign-on architectures. Single Sign-On Architectures.

[2] Hardt D. The OAuth 2.0 Authorization Framework. http://www.hjp.at/doc/rfc/rfc6749.html.

[3] Campbell B., Jones M., Mortimore C. Security Assertion Markup Language (SAML) 2.0 Profile for OAuth 2.0 Client Authentication and Authorization Grants. https://www.hjp.at/doc/rfc/rfc7522.html.

[4] Kosar T., Livny M. Stork: Making data placement a first class citizen in the grid. Proceedings of the 24th International Conference on Distributed Computing Systems.

[5] Council of European Union (2014). Council Regulation (EU) no 910/2014. https://eur-lex.europa.eu/legal-content/EN/TXT/?uri=uriserv%3AOJ.L_.2014.257.01.0073.01.ENG.

[6] Moreno R.T., Bernabe J.B., Skarmeta A., Stausholm M., Frederiksen T.K., Martínez N., Ponte N., Sakkopoulos E., Lehmann A. Olympus: Towards oblivious identity management for private and user-friendly services. Proceedings of the 2019 Global IoT Summit (GIoTS).

[7] Camenisch J., Mödersheim S., Sommer D. A formal model of identity mixer. Proceedings of the International Workshop on Formal Methods for Industrial Critical Systems.

[8] Adams C., Farrell S., Kause T., Mononen T. Internet X. 509 Public Key Infrastructure Certificate Management Protocol (CMP). http://www.hjp.at/doc/rfc/rfc4210.html.

[9] Chadwick D.W. (2009). Federated identity management. Foundations of Security Analysis and Design V.

[10] Recordon D., Reed D. (2006). OpenID 2.0: A platform for user-centric identity management. Second ACM Workshop on Digital Identity Management: DIM 2006.

[11] Langheinrich M., Abowd G., Brumitt B., Shafer S. (2001). Privacy by design—principles of privacy-aware ubiquitous systems. Ubicomp 2001: Ubiquitous Computing.

[12] Paquin C., Zaverucha G. U-Prove Cryptographic Specification v1. 1. https://www.microsoft.com/en-us/research/wp-content/uploads/2016/02/U-Prove20Cryptographic20Specification20V1.1.pdf.

[13] Sabouri A., Rannenberg K. ABC4Trust: Protecting privacy in identity management by bringing privacy-ABCs into real-life. Proceedings of the IFIP International Summer School on Privacy and Identity Management.

[14] Bernabe J.B., David M., Moreno R.T., Cordero J.P., Bahloul S., Skarmeta A. (2020). ARIES: Evaluation of a reliable and privacy-preserving European identity management framework. Future Gen. Comput. Syst..

[15] Agrawal S., Miao P., Mohassel P., Mukherjee P. PASTA: PASsword-based Threshold Authentication. Proceedings of the 2018 ACM SIGSAC Conference on Computer and Communications Security.

[16] Stadler M. Publicly verifiable secret sharing. Proceedings of the Annual International Conference on the Theory and Applications of Cryptographic Techniques.

[17] Camenisch J., Lehmann A., Neven G. Optimal Distributed Password Verification. Proceedings of the 22nd ACM SIGSAC Conference on Computer and Communications.

[18] Shoup V. Practical Threshold Signatures. Proceedings of the Annual International Conference on the Theory and Applications of Cryptographic Techniques.

[19] Langford S.K. Threshold DSS Signatures without a Trusted Party. Proceedings of the Annual International Cryptology Conference.

[20] Chaum D. Blind Signatures for Untraceable Payments. Proceedings of the CRYPTO ’82.

[21] Baum C., Frederiksen T.K., Hesse J., Lehmann A., Yanai A. (2019). Cryptology ePrint Archive, Report 2019/1470. https://eprint.iacr.org/2019/1470.

[22] Chaum D. (1981). Untraceable Electronic Mail, Return Addresses, and Digital Pseudonyms. Commun. ACM.

[23] Chaum D. (1985). Security without identification: Transaction systems to make big brother obsolete. Commun. ACM.

[24] Camenisch J., Lysyanskaya A. Cryptology ePrint Archive, Report 2001/019. http://eprint.iacr.org/2001/019.

[25] Camenisch J., Lysyanskaya A. Signature Schemes and Anonymous Credentials from Bilinear Maps. Proceedings of the Annual International Cryptology Conference.

[26] Pointcheval D., Sanders O. Short Randomizable Signatures. Proceedings of the Cryptographers’ Track at the RSA Conference.

[27] Sonnino A., Al-Bassam M., Bano S., Danezis G. Coconut: Threshold Issuance Selective Disclosure Credentials with Applications to Distributed Ledgers. Proceedings of the Network and Distributed Systems Security (NDSS) Symposium.

[28] Herzberg A., Jarecki S., Krawczyk H., Yung M. Proactive Secret Sharing Or: How to Cope With Perpetual Leakage. Proceedings of the Annual International Cryptology Conference.

[29] Camenisch J., Enderlein R.R., Neven G. Two-Server Password-Authenticated Secret Sharing UC-Secure Against Transient Corruptions. Proceedings of the IACR International Workshop on Public Key Cryptography.

[30] Camenisch J., Drijvers M., Lehmann A., Neven G., Towa P. Short Threshold Dynamic Group Signatures. https://eprint.iacr.org/2020/016.

[31] ISO/IEC CD 18013-5:2019(E) Personal Identification—ISO Compliant Driving Licence—Part 5: Mobile Driving Licence (mDL) Application. https://www.iso.org/standard/69084.html.

[32] Sakkopoulos E., Ioannou Z., Viennas E. Mobile Personal Information Exchange Over BLE. Proceedings of the 2018 9th International Conference on Information, Intelligence, Systems and Applications (IISA).

[33] Regulation (EU) no 2016/679. https://eur-lex.europa.eu/legal-content/EN/TXT/?uri=CELEX:32016R0679.

[34] Hernandez-Ramos J.L., Bernabe J.B., Skarmeta A. (2016). ARMY: Architecture for a secure and privacy-aware lifecycle of smart objects in the internet of my things. IEEE Commun. Mag..

[35] Bernal Bernabe J., Hernandez-Ramos J.L., Skarmeta Gomez A.F. (2017). Holistic privacy-preserving identity management system for the internet of things. Mobile Inf. Syst..

[36] Sanchez J.L.C., Bernabe J.B., Skarmeta A.F. (2018). Integration of Anonymous Credential Systems in IoT constrained environments. IEEE Access.

[37] Kiayias A., Zhou H.-S. Equivocal Blind Signatures and Adaptive UC-Security. https://www.iacr.org/archive/tcc2008/49480334/49480334.pdf.

[38] Jawurek M., Kerschbaum F., Orlandi C. Zero-knowledge Using Garbled Circuits: How To Prove Non-Algebraic Statements Efficiently. https://eprint.iacr.org/2013/073.

[39] Bernal Bernabe J., Canovas J.L., Hernandez-Ramos J.L., Torres Moreno R., Skarmeta A. (2019). Privacy-Preserving Solutions for Blockchain: Review and Challenges. IEEE Access.

